# The potential role of ocular and otolaryngological mucus proteins in myalgic encephalomyelitis/chronic fatigue syndrome

**DOI:** 10.1186/s10020-023-00766-8

**Published:** 2024-01-03

**Authors:** Kaylin Huitsing, Tara Tritsch, Francisco Javier Carrera Arias, Fanny Collado, Kristina K. Aenlle, Lubov Nathason, Mary Ann Fletcher, Nancy G. Klimas, Travis J. A. Craddock

**Affiliations:** 1 Department of Psychology and Neuroscience, College of Psychology, Nova Southeastern University, 3300 S. University Drive, Fort Lauderdale, FL 33328-2004 USA; 2 Institute for Neuro-Immune Medicine, Nova Southeastern University, 3300 S. University Drive, Fort Lauderdale, FL 33328-2004 USA; 3 Miami Veterans Affairs Medical Center, 1201 NW 16th St, Miami, FL 33125-1624 USA; 4 Department of Clinical Immunology, Dr. Kiran C. Patel College of Osteopathic Medicine, Nova Southeastern University, 3300 S. University Drive, Fort Lauderdale, FL 33328-2004 USA; 5 Department of Computer Science, College of Engineering and Computing, Nova Southeastern University, 3300 S. University Drive, Fort Lauderdale, FL 33328-2004 USA; 6 Present Address: Center for Collaborative Research, Room 440, Nova Southeastern University, 3300 S. University Drive, Fort Lauderdale, FL 33328-2004 USA

**Keywords:** Myalgic encephalomyelitis/chronic fatigue syndrome, Mucosal-immune system, Mucins, Inflammation, Computational modeling, Systems biology

## Abstract

Myalgic encephalomyelitis/chronic fatigue syndrome (ME/CFS) is a debilitating illness associated with a constellation of other symptoms. While the most common symptom is unrelenting fatigue, many individuals also report suffering from rhinitis, dry eyes and a sore throat. Mucin proteins are responsible for contributing to the formation of mucosal membranes throughout the body. These mucosal pathways contribute to the body’s defense mechanisms involving pathogenic onset. When compromised by pathogens the epithelium releases numerous cytokines and enters a prolonged state of inflammation to eradicate any particular infection. Based on genetic analysis, and computational theory and modeling we hypothesize that mucin protein dysfunction may contribute to ME/CFS symptoms due to the inability to form adequate mucosal layers throughout the body, especially in the ocular and otolaryngological pathways leading to low grade chronic inflammation and the exacerbation of symptoms.

## Introduction

Myalgic encephalomyelitis/chronic fatigue syndrome (ME/CFS) is a serious, long-term illness characterized by a persistent, unrelenting fatigue which is accompanied by a constellation of additional symptoms that affects many body systems. Meanwhile, the etiology of ME/CFS has yet to be fully elucidated. Among the additional symptoms tied to ME/CFS are rhinitis, sore throat, and dry eyes. Approximately, 75 to 80 percent of ME/CFS subjects appear to have an irritant rhinitis with increased mucin production (Naranch et al. 2002; Baraniuk et al. 1998), and there appears to be a relationship between ME/CFS and dry eye syndrome (Chen et al. 2018; Qanneta et al. 2014), with a previous clinical study demonstrating that sicca symptoms existed in about 70 percent of ME/CFS patients (Qanneta et al. 2014). Non-exudative pharyngitis with “crimson crescents” in the posterior pharynx is reported in upwards of 80 percent of ME/CFS patients (Cunha 1992). While other reports of related symptoms range widely (i.e. sore throat 19–84%, cervical lymphadenopathy 23–76%) it is clear that these symptoms are much more prevalent in ME/CFS as compared with healthy controls (Institute of Medicine 2015).

There are current data pointing to genetic connections of the symptoms above. A pilot study examining genome wide single nucleotide polymorphisms (SNPs) in 383 ME/CFS via the commercial company 23andMe showed that approximately 70–80% of ME/CFS subjects possess abnormal variants in genes encoding for airway, eye, and salivary mucin proteins (*MUC16*, *MUC19*, and *MUC22*) at 1.60 to 3.75 the reference population (see Table 1) (Perez et al. 2019). Many of these have the potential to generate dysfunctional mucin proteins. For example, the Combined Annotation Dependent Depletion algorithm (CADD) (Kircher et al. 2014; Rentzsch et al. 2019) indicates a maximum score of 36 for *MUC19* SNP rs10784618 followed by 24.7 for *MUC19* SNP rs11564109 where scores above 20 indicate that a particular SNP is predicted to be among the one percent most deleterious substitutions, indicating that this variant is highly deleterious, and a potential disease mitigating variant.

**Table 1:** Single Point Mutations in ME/CFS Mucin Proteins Compared to 1000 Genome Reference Population

Gene name	rsID	ME/CFS %	1000 Genome %	Ratio	Effect	Scaled CADD Score	Top % of deleterious changes	Mucin type	Location
*MUC16*	rs7245949	0.57	0.23	2.53	Missense T > I	12.17	6.09	Membrane bound	Airways, eye, reproductive organs, mesothelium
*MUC16*	rs1862462	0.50	0.25	2.02	Missense S > L	3.051	49.57		
*MUC16*	rs2547072	0.50	0.25	2.02	Missense T > I	0.635	86.41		
*MUC16*	rs1867691	0.50	0.22	2.30	Missense I > V	0.009	99.79		
*MUC19*	rs10784618	0.77	0.48	1.60	Stop_gained	36	0.03	Gel forming	Airways, eye, middle ear, salivary glands
*MUC19*	rs11564109	0.24	0.08	2.85	Missense C > Y	24.7	0.34		
*MUC19*	rs2588401	0.80	0.48	1.67	Missense A > T	7.453	18.01		
*MUC19*	rs10878538	0.26	0.11	2.33	5' UTR	4.114	38.82		
*MUC19*	rs1019709	0.82	0.40	2.05	Intron	1.241	75.17		
*MUC19*	rs2588402	0.80	0.48	1.67	Missense A > T	0.069	98.43		
*MUC22*	rs10947121	0.60	0.45	1.33	Missense L > P	5.542	27.95	Membrane bound	Airways, eye, middle ear, salivary glands
*MUC22*	rs3094672	0.75	0.20	3.67	Missense S > T	0.659	85.94		
*MUC22*	rs9262549	0.72	0.19	3.75	Missense S > T	0.27	93.98		

Mucin-19 is a secreted gel forming mucin that has been detected in the submandibular gland, sublingual gland, respiratory tract, eye, and middle ear epithelium (Linden et al. 2008). The *MUC19* SNP rs10784618 is a nucleotide change of cytosine to adenine in chromosome 12 at position 40,834,955 (GRCh37-v1.4) (Kircher et al. 2014; Rentzsch et al. 2019). This change is a coding region of the gene resulting in a nonsense point mutation causing a premature stop codon at cysteine residue 1238 in the mucin-19 amino acid protein sequence (Kircher et al. 2014; Rentzsch et al. 2019). According to UniProt (The UniProt Consortium 2021) the mucin-19 protein (UniProtKB accession number Q7Z5P9) is typically 8384 amino acids long, this mutation can result in a severely truncated, incomplete, and dysfunctional protein product that is incapable of forming a proper protective barrier. The *MUC19* SNP rs11564109 variant is a missense coding sequence variant resulting in an amino acid change at position 1411 from cysteine to tyrosine (Kircher et al. 2014; Rentzsch et al. 2019). While the significance of this alteration is uncertain, it will affect overall protein conformation as cysteine plays an important role in gel mucin structure through the formation of disulfide bonds, whereas tyrosine cannot form such bonds (Meldrum et al. 2018). The remaining variants likewise result in amino acid changes (Kircher et al. 2014; Rentzsch et al. 2019), although the consequence of the substitutions is not clear as currently the three-dimensional structure of mucin-19 is not known.

Mucin-16 and mucin-22 are both membrane-bound mucins that are present on epithelial cells and serve as receptors and sensors to mediate signal transduction. Mucin-16, known as ovarian tumor marker CA125 due to its overexpression in ovarian and endometrial cancer (Felder et al. 2014), is present in a number of normal tissues, but mainly ocular surface epithelia such as the cornea, conjunctiva, lacrimal gland, accessory lacrimal glands, efferent tear ducts and also nose, uvula and larynx (Kutta et al. 2008). Mucin-16 can restrict or facilitate microbial invasion at the apical surface of the epithelium (Chatterjee et al. 2021). For example, it has been shown that there is greater binding of *Staphlylococcus aureus* to in vitro-cultured corneal cells when mucin-16 is depleted (Blalock et al. 2007). Mucin-22 is a relatively novel membrane-bound mucin with previously unknown pathophysiological roles (Hijikata et al. 2011; Fini et al. 2020). Recent work indicates that mucin-22 appears to play an important protective role against severe coronavirus disease (COVID)-19 infection, with certain variants offering improved protection (Castelli et al. 2022). These variants in *MUC22* however did not include those observed in Table 1. Another variant in *MUC22* not listed in Table 1 appears to be associated with the risk of childhood asthma (Chen et al. 2017). Thus, while the role of ME/CFS associated variants in *MUC22* are unknown, evidence suggests they may affect the respiratory tract and response to environmental pollutants or pathogens (Castelli et al. 2022; Chen et al. 2017). The variants in Table 1 for both *MUC16* and *MUC22* result in amino acid changes in the extracellular region of the membrane bound protein. The highly glycosylated extracellular mucin domains form a tight mesh structure that protects cells by bind pathogens to inhibit invasion (Putten and Strijbis 2017). It is known that serine and threonine repeats in this region are the sites of glycosylation (Brown et al. 2013), thus if these variants produce functional changes in these mucin proteins, it would therefore be in the ability of these mucins to interact with and bind pathogens.

## The hypothesis

**Fig. 1:**
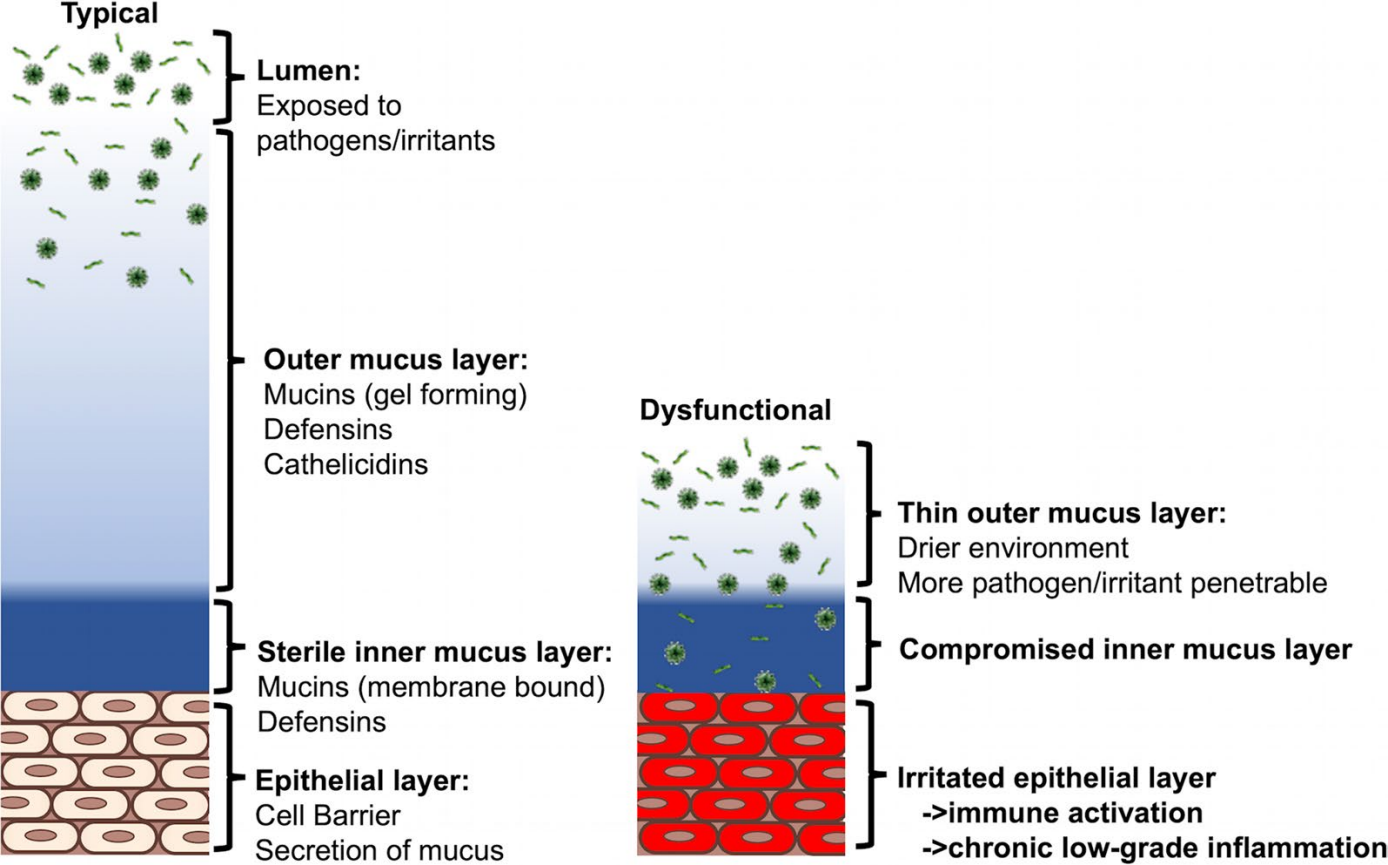
The mucosal protective barrier. (Left) Pathogens present in the lumen are prevented from reaching the epithelium via a first layer of gel-like mucins and antibacterial peptides, and a second layer of membrane bound mucins (Linden et al. 2008; Dhanisha et al. 2018). (Right) Dysfunctional outer and inner mucin proteins lead to a compromised inner mucus layer and irritated epithelium (Linden et al. 2008; Dhanisha et al. 2018)

The role of these mucus proteins (mucin-16, mucin-19 and mucin-22) in the ear, nose, throat, respiratory tract, and eye is to protect and prevent infection (Linden et al. 2008; Dhanisha et al. 2018). Exposure to the environment risks exposure to viral and bacterial pathogens (Fig. 1). The first layer of protection from these pathogens is the outer mucus layer formed of gel like mucins (such as mucin-19), and anti-bacterial peptides such as defensins and cathelicidins (Linden et al. 2008; Dhanisha et al. 2018; Repentigny et al. 2015). This outer mucus layer serves to protect the second inner mucus layer adhered to the epithelium, keeping it sterile to avoid irritating the epithelial layer (Fig. 1, left). Dysfunctional changes that decrease the outer gel layer of mucus (i.e., through non-functional mucin-19), coupled with an inner mucus layer incapable of binding pathogens (i.e., dysfunctional mucin-16 and mucin-22) would result in a compromised mucus layer leading to a chronically irritated epithelial layer (Fig. 1, right).

**Fig. 2:**
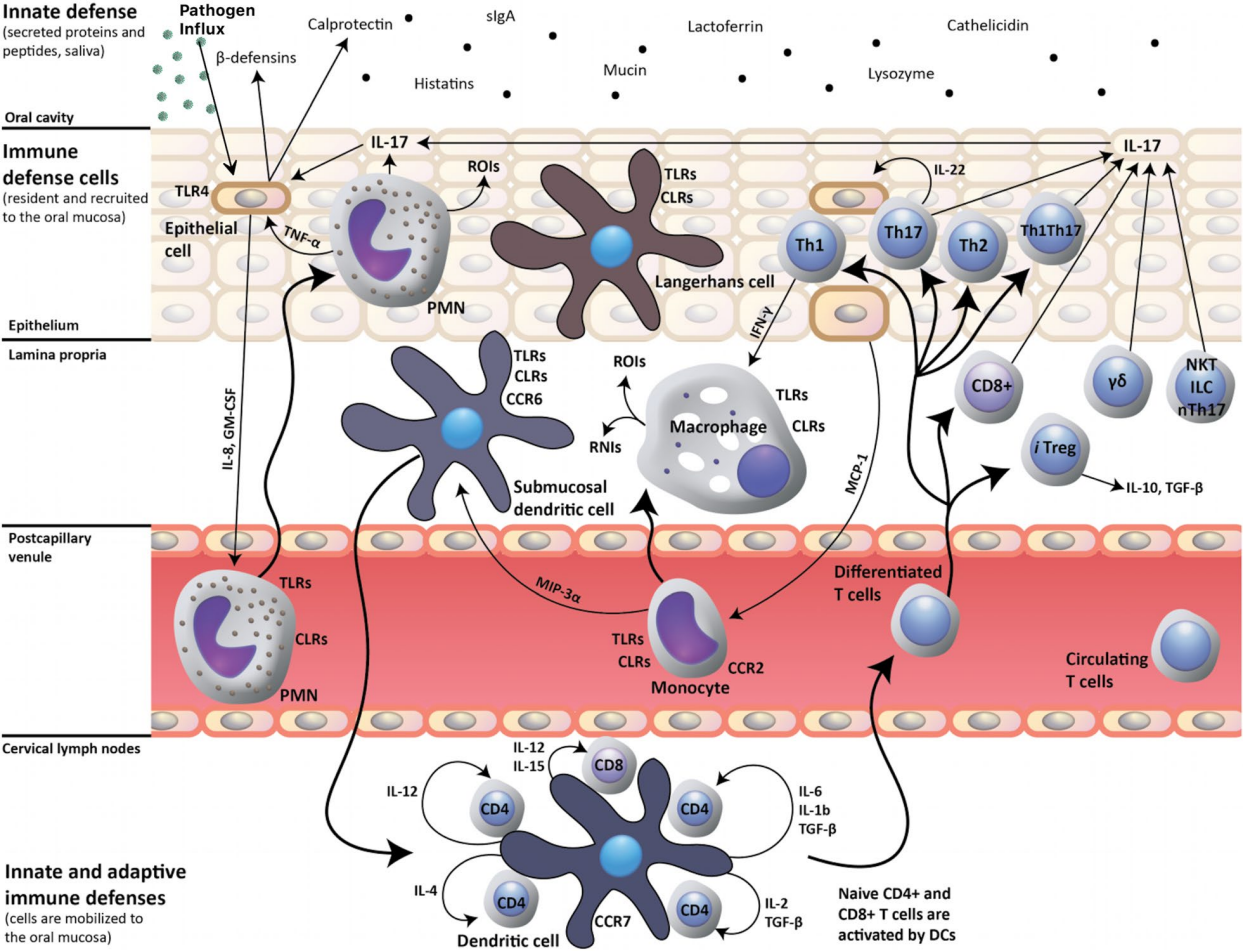
Host response to oral pathogen influx. A protective host response to infection is dependent on dendritic cell-mediated induction of Th17 cell-mediated adaptive immunity, which, by the production of interleukin (IL)-17 upregulates the innate expression of mucosal antimicrobial peptides (β-defensins, calprotectin) by epithelial cells. IL17 also up-regulates IL8 and granulocyte–macrophage colony-stimulating factor (GM-CSF) production by epithelial cells, which in turn trigger recruitment of polymorphonuclear neutrophils (PMN) to the oral mucosa. Innate-like cell populations, including γδ T-cells, Natural Killer T cells (NKT), innate lymphoid cells (ILC) and natural Th17 cells (nTh17), also produce IL17 and may participate in the mucosal host response. CLRs, C-type lectin receptors; RNIs, reactive nitrogen intermediates; ROIs, reactive oxygen intermediates; and TLRs, toll-like receptors. Image adapted from (Repentigny et al. 2015) under the Creative Commons Attributions License 4.0 International (CC BY 4.0)

When pathogens manage to cross the mucus layer the response by the immune system from resident and recruited immune defense cells containing T-cells, dendritic cells, and macrophages, and further from the immune system within the blood comprising of peripheral blood mononuclear cells (Fig. 2) would result in a chronic low-grade inflammation (Repentigny et al. 2015; Zhang et al. 2023; Song et al. 2020). Expanding on the roles of the mucosa and cytokines within the ocular and otolaryngological environment, there is speculation as to the resulting deficits in the mucin-16, mucin-19 and mucin-22 proteins. While preliminary studies have shown that numerous individuals have variants in these proteins (1000 Genomes Project Consortium (2015), it is hypothesized that these variants cause dysfunction in the mucosal protective barrier. A dysfunctional mucosal barrier will result in a compromised barrier between epithelial cells and the environment. Smaller or weaker barriers permit pathogen access and infiltration leading to a persistent low-grade inflammation in which cytokines will be consistently released contributing to continuous sickness behaviors like those seen with ME/CFS.

## Evaluation of the hypothesis

### Discrete ternary logic analysis of regulatory network

**Fig. 3:**
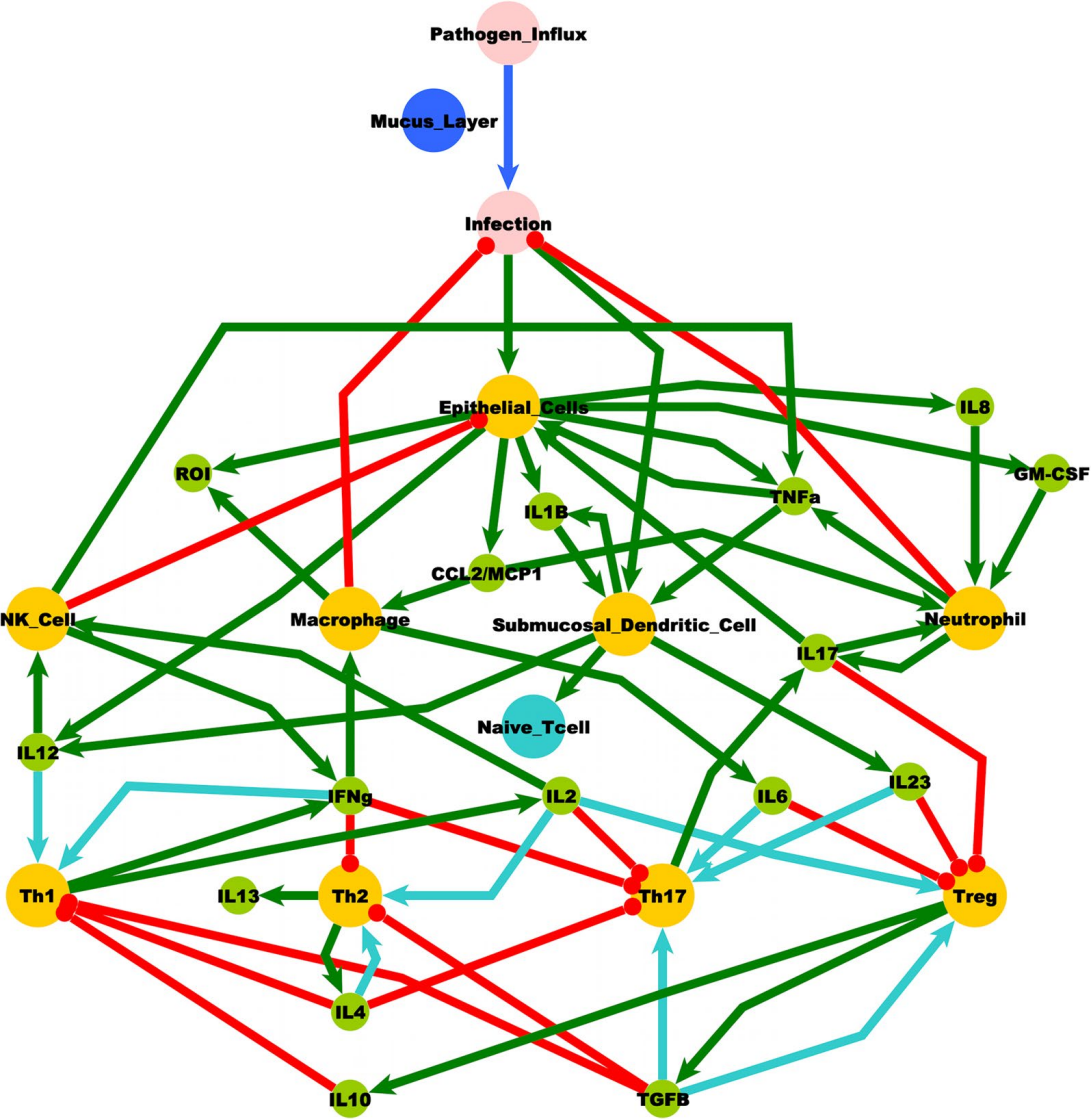
A literature-based logic model of mucosal-immune system interactions. Nodes: Yellow, distinct immune cell types; Green, immune signaling molecules; Red, environmental pathogens. Edges: Red, inhibition; Green, stimulation. Conditionals: Cyan, Naïve T Cell dependent stimulation; Blue, Mucus layer dependent stimulation. GM-CSF—granulocyte–macrophage colony-stimulating factor; IFNg–interferon γ; IL–interleukin; ROI–reactive oxygen intermediary; NK–natural killer cells; CCL2/MCP1—chemokine ligand 2/monocyte chemoattractant protein 1; TGFB–transforming growth factor β; Th–T helper cell; TNFa–tumor necrosis factor α; Treg–T regulator cell

Our previous work (Craddock et al. 2014, 2015, 2018; Fritsch et al. 2014) suggests that the complexity of the mucosal-immune signaling system can allow for multiple regulatory modes beyond what is typically considered typical health. To provide a theoretical framework for our hypothesis here we compile molecular and cellular signaling information from various studies and reviews in the literature (Repentigny et al. 2015; Ohradanova-Repic et al. 2023; Romero et al. 2004; Kato 2015; Barnes et al. 2022; Mettelman et al. 2022; Sato and Kiyono 2012; Davis et al. 2017; Costello et al. 2015; Laulajainen-Hongisto et al. 2020) to create a logically consistent, theoretical model of a general ocular and otolaryngological mucosal-innate immune signaling system to explore the role of mucus protection in the homeostatic regulation of the innate immune system and the perpetuation of chronic low-grade inflammation (see Fig. 3). Logic rules are applied to this connectivity diagram to predict the system’s homeostatic behavior. Using a similar approach reported in (Craddock et al. 2015; Craddock et al. 2014; Thomas 1991; Thomas et al. 1995; Mendoza and Xenarios 2006) the mucosal-immune system in Fig. 3 was captured as a logic connectivity model consisting of interconnected nodes with three discrete states: − 1 (suppressed), 0 (normal) and + 1 (increased). In brief, the state of the system at a point in time was described by an assignment of discrete states to all nodes. The state that each node in the system transitions to in the next time step was determined from a set of balanced ternary logic statements [see (Craddock et al. 2015)], the node’s current state, and the defined interactions (i.e. activate or inhibit) of the neighboring input nodes. The logic is such that an increase in activators raises the node value, while a decrease in inhibitors decreases the value. In cases where both activators and inhibitors were increased, the node value remained unchanged. While the number of activators and/or inhibitors for a given variable may remain static, they may also be allowed to change based on predefined conditions, such as the state of one or more variables as described in Arias et al. (2021). In the system described in Fig. 3 conditional edges are dependent on the state of Naïve T Cells. The system is updated asynchronously (allowing only one variable to change at a time), such that for each current state there are potentially several subsequent states towards which it may evolve. The number of states, and the values they can be assigned, determine the total number of states available to the model system. By analyzing all possible states of the system, a temporal sequence of states was discerned. Steady states were defined as those states for which the current system state did not evolve in time. The steady states of the mucosal-immune system are given in Table 2.

**Table 2:** Stable behaviors of the mucosal-immune system

Nodes	SS0	SS1	SS2
Submucosal dendritic cell	0	1	1
Infection	0	0	0
Epithelial cell	0	0	− 1
IL1β	0	1	1
TNFα	0	1	0
IL12	0	1	1
IL8	0	0	− 1
CCL2/MCP1	0	0	− 1
GM-CSF	0	0	− 1
Naive Tcell	0	1	1
Th1	0	1	1
Th2	0	0	− 1
Th17	0	0	− 1
Treg	0	0	− 1
IFNγ	0	1	1
IL2	0	1	1
IL6	0	1	1
TGFβ	0	0	− 1
IL4	0	0	− 1
IL10	0	0	− 1
IL23	0	1	1
IL17	0	1	0
IL13	0	0	− 1
Neutrophil	0	1	0
ROI	0	1	1
Macrophage	0	1	1
NK cell	0	1	1

Beyond normal homeostatic regulation, our model predicts alternate self-perpetuating conditions consistent with chronic inflammation. Three stable states are shown in Table 1 with SS0 corresponding to a typical healthy state, while both SS1 and SS2 present with a stable altered Th1 immune profile. As such, these simulations of pathogen influx with deficient mucus protection were shown to be theoretically capable of forcing the system to a state of immune activation supporting a potential role for the mucosal-immune signaling system’s own homeostatic drive in perpetuating chronic low-grade inflammation.

### Comparison to ME/CFS cytokine panels

**Fig. 4:**
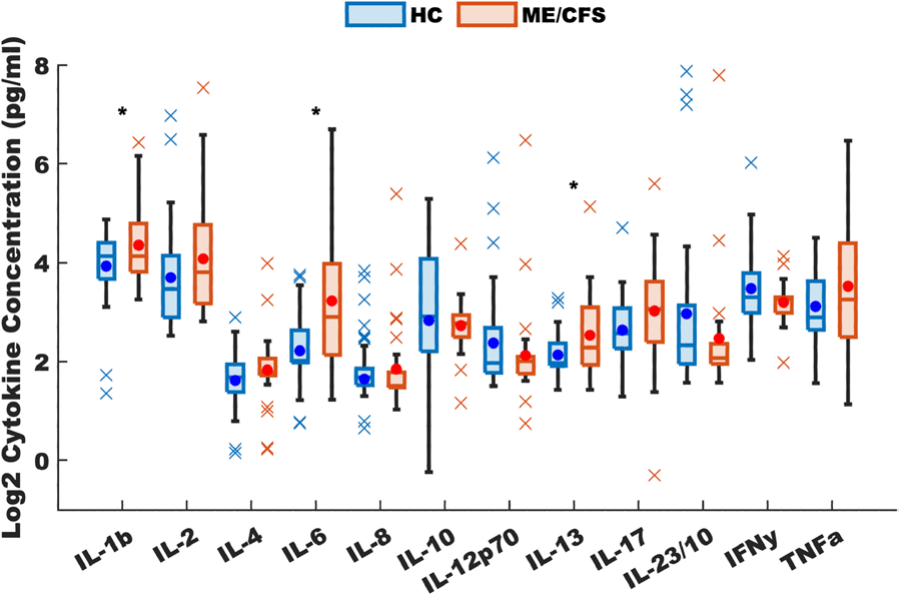
Log2 normalized plasma cytokine concentrations at rest for female ME/CFS subjects compared to healthy sedentary controls. Horizontal line indicates the median, solid dot indicates the overall mean, box shows the lower and upper quartiles, x indicates outliers, and whiskers indicate the minimum and maximum values that are not outliers. *p < 0.05 for two-tailed heteroscedastic t-test. Note: IL23 concentration is reduced by a factor of 10 to fit on the graph scale

**Fig. 5:**
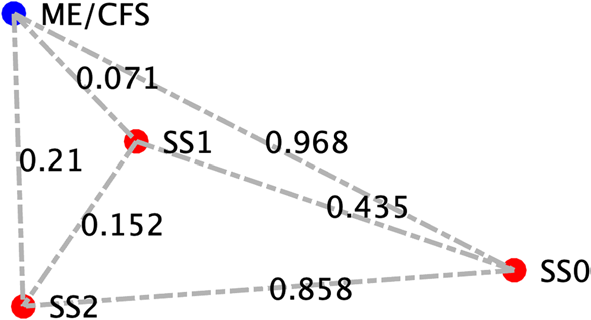
Projections of the comparison between the model predicted stable states and female ME/CFS cytokine profiles. SS0 indicates typical health, SS1 is a stable state with an increased Th1 immune profile, and SS2 is a stable state with an increased Th1 and decreased Th2, Th17 and Treg immune profile. Distances between points are measures of dissimilarity as determined by an aggregate P-value based on Brown’s methods for combining multiple non-independent statistical tests

**Fig. 6:**
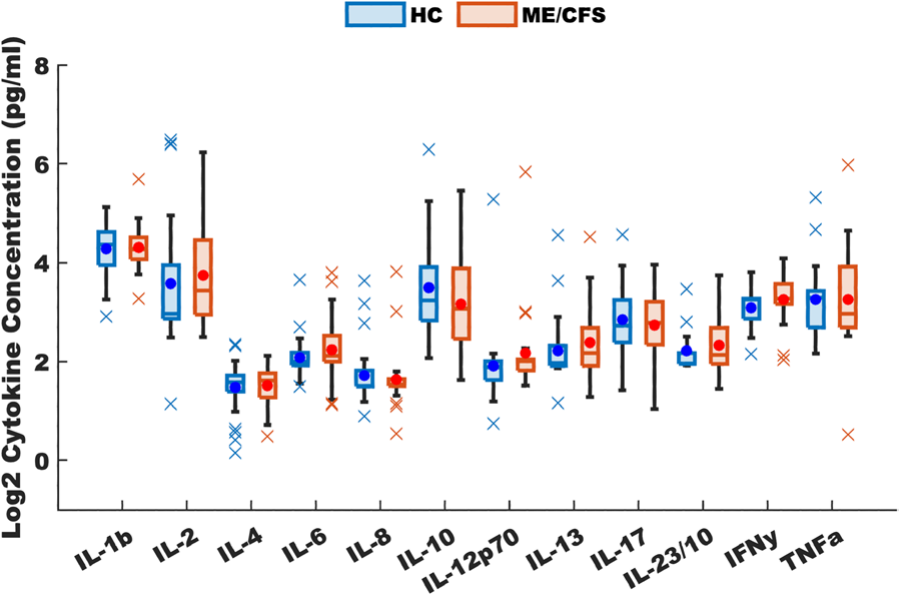
Log2 normalized plasma cytokine concentrations at rest for male ME/CFS subjects compared to healthy sedentary controls. Horizontal line indicates the median, solid dot indicates the overall mean, box shows the lower and upper quartiles, x indicates outliers, and whiskers indicate the minimum and maximum values that are not outliers. *p < 0.05 for two-tailed heteroscedastic t-test, no significant differences found. Note: IL23 concentration is reduced by a factor of 10 to fit on the graph scale

**Fig. 7:**
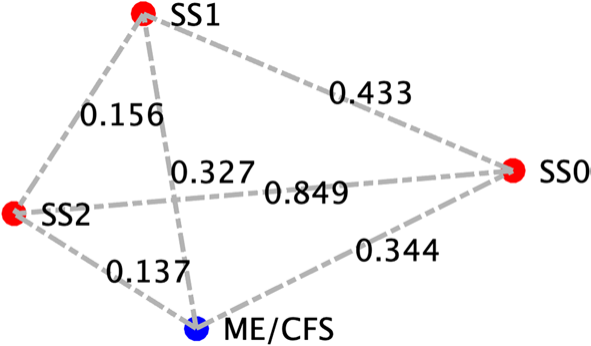
Projections of the comparison between the model predicted stable states and male ME/CFS cytokine profiles. SS0 indicates typical health, SS1 is a stable state with an increased Th1 immune profile, and SS2 is a stable state with an increased Th1 and decreased Th2, Th17 and Treg immune profile. Distances between points are measures of dissimilarity as determined by an aggregate P-value based on Brown’s methods for combining multiple non-independent statistical tests

To determine the applicability of this model to ME/CFS in specific, we compared our model predicted homeostatic stable states to cytokine signaling profiles in blood of female subjects with ME/CFS. Clinical data obtained as part of a larger on-going study investigating changes in cytokines in ME/CFS was used as a basis for comparison with the predicted resting states [see (Morris et al. 2019)]. A total of 65 female subjects (29 with ME/CFS, 36 healthy controls), and 53 male subjects (25 with ME/CFS, 28 healthy controls) were selected without exclusion for ethnicity from the patient population within the Institute for Neuroimmune Medicine at Nova Southeastern University (NSU) in Fort Lauderdale, Florida, directed by Nancy Klimas, M.D. All subjects signed an informed consent approved by the Institutional Review Board (IRB) of NSU, Fort Lauderdale, Florida. Included subjects presented with acute onset and with an illness duration of at least 4 years. ME/CFS was diagnosed according to current research case definitions (Fukuda et al. 1994; Carruthers et al. 2003): fatigue of greater than 6 months duration and at least four of eight symptoms including exercise-induced relapse, myalgia, arthralgia, headache of a new and different type, nonrestorative sleep, cognitive complaints, sore throat, and tender lymph nodes. All ME/CFS study subjects presented with a 36-short form health survey (SF-36) summary physical composite score below the 50th percentile, based on population norms. Healthy controls were self-defined as sedentary (no regular exercise program, sedentary employment). Plasma concentrations of interleukin (IL) 1β, IL2, IL4, IL6, IL8, IL10, IL12p70, IL13, IL17, IL23, IFNγ and TNFα were measured via Q-Plex multiplex ELISA (Quansys Biosciences, Logan, Utah) from blood obtained at rest. A meta-analysis was used to calculate the significance of similarity between the inflammatory profiles of subjects with ME/CFS, and the equilibrium states predicted by the logic model. To do this the cytokine profiles were compared to each model-predicted steady-state behavior of the mucosal-immune system through the application of Brown’s theoretical approximation (Brown 1975) of Fisher’s statistics, as conducted in our previous work (Craddock et al. 2014, 2015, 2018; Fritsch et al. 2014; Arias et al. 2021; Rice et al. 2014). This method was chosen as it provides a meta-analysis technique to combine non-independent probabilities and obtain an overall significance measure P based on a set of *p*-values obtained from independent *t*-tests. The aggregate value P ranges between 0 and 1, with 0 indicating complete overlap and 1 being the farthest distance from a stable state. This method is applicable as the model elements do not express independently, as evidenced by the connectivity of the mucosal-immune interaction model (Fig. 3). The above-mentioned cytokine data were compared against the model predicted states based on the 12 measured variables. To visualize the comparison of the measured states with the model-predicted stable states the multi-dimensional co-expression profiles (Figs. 4 and 6) were projected into a two-dimensional space using multidimensional scaling as done previously (Arias et al. 2021) (see Figs. 5 and 7). Here, the dissimilarity matrix defined by the aggregate P value is scaled such that the 2D Euclidean distances between points approximate the corresponding dissimilarities. This is performed using the function *mdscale* in MATLAB to minimize Kruskal’s stress criterion normalized by the sum of squares of the dissimilarities. After comparing the stable states in Table 2 with the female cytokine profiles of ME/CFS patients (Fig. 4), we found that SS1 was the most closely aligned with the ME/CFS profile (Fig. 5). The SS1 state is characterized as an inflammatory state by increased pro-inflammatory cytokines (IL1β, IL2, IL6, IL12, IL17, IL23, IFNγ, TNFα), activation of the innate immune cells (Neutrophil, Macrophage, NK Cell, Submucosal Dendritic Cell) and a shift towards Th1 immunity. The next nearest state was the SS2 state. The SS2 state is characterized as a mixed inflammatory state by an increase in some pro-inflammatory cytokines (IL1β, IL2, IL6, IL12, IFNγ), a decrease in other pro-inflammatory cytokines (IL8, IL23, TNFα), a decrease in anti-inflammatory cytokines (IL4, IL10,TGFβ), activation of some of the innate immune cells (Macrophage, NK Cell, Submucosal Dendritic Cell), an upward shift towards Th1 and downward shift away from Th2, Th17, and Treg immunity. Female ME/CFS cytokine profiles were furthest from the typically healthy state SS0. This is consistent with our hypothesis that ME/CFS presents with a low-grade inflammatory profile. However, when comparing the male cytokine profile of ME/CFS subjects (Fig. 6), it was found to align near equidistant from health (SS0) and the SS1 state, with the SS1 being slightly more favorable (Fig. 7), but unlike females it was found to be the closest to the SS2 mixed inflammatory state. The difference between male and female cytokine profiles is consistent with previous work suggesting a sex difference in males with ME/CFS (Jeffrey et al. 2019; Nkiliza et al. 2021; Friedberg et al. 2023; Lim and Son 2021; Cheema et al. 2020; Pollack et al. 2023).

## Consequences of the hypothesis and discussion

The ocular and otolaryngological mucus layers normally act to protect the epithelial tissue from irritants, microorganisms and pathogens entering the body (Linden et al. 2008; Dhanisha et al. 2018). Changes in the mucus layer lining can often be symptoms of illness such as diabetes (Negrato and Tarzia 2010), human immunodeficiency virus (HIV) (Heron and Elahi 2017), vitamin deficiency (Philipone et al. 2017) or even neurodegenerative illnesses such as Alzheimer’s and Parkinson’s diseases (Auffret et al. 2021; Paraskevaidi et al. 2020). Here we have presented a hypothesis that the symptoms observed in the chronic illness of ME/CFS may, in part, be associated with a compromised ocular and otolaryngological mucus layer leading to increased likelihood of irritation of the epithelial layer in these regions resulting in a chronic low-grade inflammation. This is consistent with findings indicating a preponderance of rhinosinusitis symptoms in subjects with unexplained chronic fatigue and bodily pain (Chester 2003).

The alignment of several immune markers modeled here with experimental data from men and women with ME/CFS supports at least partial involvement of the body's own homeostatic drive in facilitating the perpetuation of this condition and a chronic dysregulated immune system. When considering these alignments, however, it is important to remember that the hypothesis outlined above does not state that ME/CFS results solely from inflammation. Instead, it is proposed that homeostatic drive might be a significant contributor to the persistence of illness mechanisms, which may be exacerbated by a compromised mucosal protective barrier. These naturally occurring alternate immune regulatory regimes, once entered, provide an alternate stable homeostasis resistant to change, that can support many chronic pathological processes (Craddock et al. 2014; Fritsch et al. 2014), including ME/CFS symptoms related to inflammation (Komaroff 2017). This alignment is also not expected to remain static (Fritsch et al. 2014). Under typically healthy conditions small perturbations of the immune system result in a regulatory response that will fluctuate in time around its normal homeostatic state (Fritsch et al. 2014). Likewise, an illness in the vicinity of an alternate stable state will also fluctuate, albeit differently from health (Fritsch et al. 2014). This is consistent with literature findings of mixed and variable inflammation profiles in ME/CFS (VanElzakker et al. 2019; Blundell et al. 2015; Strawbridge et al. 2019). It is this overall altered inflammation regulation which can correlate with symptoms in ME/CFS (Komaroff 2017), even though static cytokine profiles themselves are transient and variable.

Beyond the potential exacerbation of immune activation caused by a compromised mucus layer it is difficult to discuss the consequences of these mucin variants without knowing if they are over/under expressed in ME/CFS. The GWAS SNP analysis presented here only exposes the presences of these variants in the illness cohort. However, past studies have shown immune, and brain functional consequences associated with the mucin SNPs found in ME/CFS as presented in Table 1. For the *MUC16* SNP rs7245949 a study of the association with tumor mutation burden has shown it is associated with lower expression in markers of T-cell responses (Wang et al. 2020). The remaining three *MUC16* SNPs (rs1862462, rs1867691, rs2547072) have all been shown to be significantly associated with changes in grey matter volumes in ten brain regions in a study of genetic biomarkers for Alzheimer’s Disease (Zeng et al. 2021). Two of the *MUC19* SNPs (rs2588401and rs2588402) have been associated with the chronic inflammatory disorder asthma (Karunas et al. 2015a, b; Levchenko et al. 2018), while *MUC19* SNP rs2588401 shows an association with acute lymphocytic leukemia, a cancer of immature lymphocytes, a type of white blood cell involved in the body's immune system (Alcântara et al. 2022). The *MUC22* SNP rs10947121 has been associated with ankylosing spondylitis, a type of arthritis that causes inflammation in the joints and ligaments of the spine, as well as peripheral joints like the knees, ankles, and hips (Chen et al. 2016). Finally, an association between *MUC22* SNP rs3094672 and the autoimmune disorder multiple sclerosis has been shown, albeit with a protective role (Dankowski et al. 2015). To the best of our knowledge, beyond these handful of studies, the functional consequences of the mucin variants identified here in ME/CFS have not been investigated.

The association of these variants with inflammatory disorders may point to a role in ME/CFS. Many studies of ME/CFS have found evidence of abnormal T cell populations (Mandarano et al. 2020; Rivas et al. 2018; Brenu et al. 2016; Fletcher et al. 2000), and reduced natural killer (NK) cell function (Barker et al. 1994; Whiteside and Friberg 1998; Fletcher et al. 2002; Brenu et al. 2012, 2014; Klimas et al. 1990). Studies indicate NK cell function correlates with illness severity (Rivas et al. 2018; Ojo-Amaize et al. 1994; Strayer et al. 2015), however the reason for this reduced function is unknown. As T and NK cells need to form immune synapses with their target which involve very close cell–cell contact (Bromley et al. 2001) the presence of an adhesive or anti-adhesive molecule, like mucins, on the surface of cells may have significant consequences for cell interactions. As discussed above, *MUC16* SNPs associated with ME/CFS are associated with T cell and lymphocyte dysfunction. Additionally, mucin-19 is suggested to specifically alter CD4 + T cell response (McBride et al. 2020). It’s hypothesized that this dysfunction is due to the large and heavily glycosylated mucin-16 preventing the establishment of a robust immunologic synapse between T cells and major histocompatibility complex presentation of antigens on the cell surface (Wang et al. 2020). This is supported by flow cytometry results which suggest significant deficits in the expression of receptors and adhesion molecules on subsets of CD8 + T cells in ME/CFS patients may contribute to disease pathogenesis (Brenu et al. 2016). Moreover, mucin-16, either detached or adhered to the cell surface, has been shown to directly inhibit the natural cytotoxicity mechanism of NK cells (Gubbels et al. 2010). NK cell activation triggered by inflammatory mediators, cytokines, and chemokines, including IL-2 and IL-12 following recognition of stressed, and infected cells leads NK cells to lyse target cells and secrete IFNγ and TNFα (Hardcastle et al. 2015). The reduced ability of NK cells to recognize and clear infected and stressed epithelial cells coupled with the proposed increased propensity of the epithelium to be irritated and infected in ME/CFS due to dysfunction in the mucus layer is expected further exacerbate this problem leading to an increase in associated symptoms with a decrease in NK cell function consistent with literature.

While studies of the direct functional consequences of ME/CFS identified mucin SNPs are lacking, the consequences of a dysfunctional mucus barrier also have additional relevance for ME/CFS and its symptomatology. For example, chemical sensitivities are recognized as a common symptom of ME/CFS (Carruthers et al. 2003, 2011) with multiple chemical sensitivity being a common comorbidity in the illness (Carruthers et al. 2003; Reid 1999). Triggers include pesticides, perfume and petrochemicals, and natural irritants like mold and wood-fire smoke, and can lead to symptoms of headache, migraine, cognitive impairment, dizziness, fatigue, nausea, vomiting, cardiac abnormalities, skin rashes, asthma, and anaphylaxis (Damiani et al. 2021) all of which are common symptoms of ME/CFS. A dysfunctional ocular and otolaryngological mucus layers would lead to a sensitive epithelium that may be irritated due to environmental exposures (i.e. chemical or biological) leading to “flares” of symptoms as the immune system is further triggered. This is consistent with the “kindling” theory of ME/CFS (Jason et al. 2009). ME/CFS has also been associated with exposure to infectious agents, and there have been multiple reported “outbreaks” of illness (Monro and Puri 2018). Various bacteria, including members of the gut microbiome, and viruses such as human parvovirus B19, enteroviruses, as well as the herpesviruses Epstein–Barr virus (EBV), human herpesvirus-6 types A and B (HHV-6), and human cytomegalovirus (HCMV), have been implicated as possible etiological pathogens of ME/CFS (Ariza 2021; Cox et al. 2022). The symptom similarities between Long COVID (post-acute sequela of SARS-COV-2 infection) and ME/CFS also suggest that COVID-19 may play a similar role in disease onset (Komaroff and Lipkin 2023). These pathogens are all found in, and can be transmitted by, saliva or respiratory droplets. A compromised otolaryngological mucus layer would allow for increased risk of initial infection. Furthermore, the herpes viruses (i.e. EBV, HHV-6, and HCMV) remain latent within the body within salivary glands and epithelial cells and occasionally are reactivated (Grinde 2013). Potential triggers of this reactivation include environmental irritants leading to inflammation (Stoeger and Adler 2019; Williams et al. 2019). As such, increased irritation in these regions due to a dysfunctional mucus protective barrier would lead to a greater incidence of viral reactivation and its associated symptoms.

Finally, similar to our findings presented here, many studies indicate a sex difference in ME/CFS in symptom presentation, onset and triggers (Friedberg et al. 2023; Thomas et al. 2022), immune measures (Jeffrey et al. 2019; Cheema et al. 2020; Smylie et al. 2013), metabolism (Nkiliza et al. 2021; Thomas et al. 2022), and gut microbiota (Wallis et al. 2017, 2018) with the female sex at greater risk for developing the illness and suffering endocrine events over the illness course (Pollack et al. 2023; Thomas et al. 2022). Females with ME/CFS report greater irregularities in their menstrual cycles, with menopause and pregnancy affecting their symptomatology (Pollack et al. 2023). Endometriosis, a disease in which tissue similar to the lining of the uterus grows outside the uterus, is a comorbid condition in women with ME/CFS, and is associated with chronic pelvic pain, earlier menopause, hysterectomy, and more ME/CFS-related symptoms (Wirth and Löhn 2023). While the mucins identified here in ME/CFS cluster in the ocular and otolaryngological areas, mucin-16 is also present in the endometrium with its overexpression in ovarian and endometrial cancer denoting it as a known ovarian tumor marker (Felder et al. 2014). Serum levels of mucin-16 are increased in women with endometriosis, however no changes have been found in the expression of mucin-16 in the endometrium of women with endometriosis (Liu et al. 2020). In ovarian cancer, mucin-16 is reported to be an immunosuppressive factor by acting on the surface of NK cells, B cells, monocytes, and neutrophils leading to an inflammatory and immunosuppressive phenotype (Wu et al. 2023). As such increased levels of mucin-16 in serum would additionally add to an altered immune profile in ME/CFS. As it has also been shown that dexamethasone, as a synthetic steroid similar to the stress hormone cortisol, upregulates the expression of mucin-16, this provides an additional link between mucin-16 expression, stress, and immune dysregulation in ME/CFS (Seo et al. 2007; Karlan et al. 1988). Should the variants in *MUC16* noted here result in a dysfunctional mucus that further contributes to these changes in inflammation seen in ME/CFS it may explain the overall sex differences and additional symptoms observed in females. Further investigation however is needed.

Overall, we have presented genetic evidence suggesting a dysfunctional mucus barrier in most individuals with ME/CFS. This dysfunction, in conjunction with environmental exposures to chemical and biological triggers, potential latent viral infection, altered T and decreased NK cell function are expected to contribute to the overall triggering of symptoms in ME/CFS. Future work investigating the role of the ocular and otolaryngological mucus layer are ultimately needed to confirm this hypothesis.

## Data Availability

The datasets used and/or analyzed during the current study available from the corresponding author on reasonable request.

## Abbreviations

CADD: Combined annotation dependent depletion algorithm
CCL2/MCP1: Chemokine ligand 2/monocyte chemoattractant protein 1
CLRs: C-type lectin receptors
COVID: Corona virus disease
EBV: Epstein–Barr virus
GM-CSF: Granulocyte–macrophage colony-stimulating factor
HCMV: Human cytomegalovirus
HHV6: Human herpesvirus-6 types A and B
IL: Interleukin
ILC: Innate lymphoid cells
ME/CFS: Myalgic encephalomyelitis/chronic fatigue syndrome
NK: Natural killer
NKT: Natural killer T
NSU: Nova Southeastern University
nTh17: Natural Th17 cells
PMN: Polymorphonuclear neutrophils
RNIs: Reactive nitrogen intermediates
ROIs: Reactive oxygen intermediates
SF-36: 36-Item short form health survey
SNP: Single nucleotide polymorphisms
SS: Steady state
TGFB: Transforming growth factor β
Th: T helper cell
TLRs: Toll-like receptors
TNFa: Tumor necrosis factor α
Treg: T regulator cell
